# Eye tracking applied to tobacco smoking: current directions and future perspectives

**DOI:** 10.16910/jemr.15.1.2

**Published:** 2022-01-21

**Authors:** Matteo Valsecchi, Maurizio Codispoti

**Affiliations:** Universitá di Bologna, Dipartimento di Psicologia

**Keywords:** smoking, eye tracking, exploration, salience, graphic warning label

## Abstract

Over the years the general awareness of the health costs associated with tobacco smoking has motivated scientists to apply the measurement of eye movements to this form of addiction. On one hand they have investigated whether smokers attend and look preferentially at smoking related scenes and objects. In parallel, on the other hand eye tracking has been used to test how smokers and nonsmokers interact with the different types of health warning that policymakers have mandated in tobacco advertisements and packages. Here we provide an overview of the main findings from the different lines of research, such as the evidence related to the attentional bias for smoking cues in smokers and the evidence that graphic warning labels and plain packages measurably increase the salience of the warning labels. We point to some open questions, such as the conditions that determine whether heavy smokers exhibit a tendency to actively avoid looking at graphic warning labels. Finally we argue that the research applied to gaze exploration of warning labels would benefit from a more widespread use of the more naturalistic testing conditions (e.g. mobile eye tracking or virtual reality) that have been introduced to study the smokers’ attentional bias for tobacco-related objects when freely exploring the surrounding environment.

## Tobacco smoking: Health and Policy

An analysis of the data on the global tobacco epidemic based from the Global Burden of Disease Study 2019 (84) testifies to the persistent relevance of this problem at the world level. As of 2019, tobacco smoking was still responsible for approximately 7.69 million deaths a year. The global prevalence of tobacco smoking among individuals aged 15 years or older was estimated at 32.7% among males and 6.62% among females. Although the prevalence of smoking among adults has decreased on a global level between 1990 and 2019 by 27.5% among males and by 37.7% among females, the number of smokers has increased for most of the two decades considered, due to the global growth in the world population. Tobacco consumption also extends beyond smoking. As of 2019 in the United States approximately 20.8% of adults reported consuming any tobacco product. A considerable amount of them (19.5% of consumers) were not consuming combustible products (19).

Policy makers in different countries have adopted various strategies to reduce tobacco consumption, including bans on advertising tobacco products and mandatory warnings on tobacco product packs. These are however far from complete, so that, as from the World Health Organization Report (103), in 2018 a large share of the world population (82%) was still not covered by the maximum level of advertisement ban, and mandatory pack warnings were only covering 52% of the world population. This means that a considerable share of the world population is still exposed to stimuli that are meant to increase their likelihood of smoking, as well as stimuli that are meant to have the opposite effect. In some contexts, this can happen even at the same time, as is the case in the countries where legislation mandates those warnings to be applied to tobacco advertisements.

Given the relevance of the health hazard posed by tobacco consumption, the relatively wide availability of smoking-related material in everyday environments, at least compared to illegal addictive substances, and given the widespread use of visual stimuli in campaigns that are meant to reduce smoking, it is not surprising that over the years eye tracking has been extensively applied to understanding how smokers interact with smoking related visual material.

## Three main approaches in the application of eye-tracking to smoking

In this review we provide an overview of the different approaches and experimental designs that have been employed in smoking-related eye-tracking studies, summarize the main findings and provide suggestions about possible developments in this field of research. To make it easier for the reader to get a structured overall picture, we identify three main ways in which eye tracking has been applied to smoking-related material, which we will present separately in the following paragraphs. The first relates to studies which have used eye-tracking to investigate the attentional bias that smokers might have towards smoking-related visual content when it competes with smoking-unrelated content. The second category refers to studies that have been conducted to evaluate how smokers react to the presence of smoking-related content in a scene or in the environment, with the aim of understanding how tobacco addiction modifies oculomotor behavior in a naturalistic context. The third category of studies relates to the use of eye tracking to evaluate the effectiveness of the stimuli which are explicitly designed to either promote tobacco products or to dissuade the viewer from the consumption of tobacco, i.e. advertisements or warnings. In the last section we discuss what we see as the main achievements and the current open questions and limitations in this field, in particular we advocate for a stronger integration of naturalistic approaches in the research aiming at evaluating the effectiveness of anti-smoking warnings.

In the review we focus specifically on the use of eye tracking applied to the way smokers and nosmokers explore tobacco-related material. We do not delve into other related topics, such as the effect of nicotine on the oculomotor system per se, the neurobiology of addiction, the different factors that can promote craving, and eye movements in different types of addiction. Although we focus on tobacco, some of the findings that we highlight are relevant to other addictive substances, especially those that are legally sold and advertised while being targeted by institutional dissuasive or health information campaigns, such as alcohol or unhealthy foods in some contexts.

## Eye tracking as a measure of attentional bias towards smoking related cues

Multiple theories of addiction posit that individuals with dependence should pay preferential attention to smoking-related cues. This could happen because they become hyper-sensitized to drug-related stimuli, which have been attributed ‘incentive salience’ (86), or because drug-related stimuli have become automatically associated with drug-related action schemata (94).

One way in which this prediction has been investigated is to expose people with and without dependence, in particular smokers and nonsmokers in the context of this review, to drug-related content in spatial competition with drug-unrelated content, to verify whether drug-related content acts as an attentional cue. This approach traces its origins back to the work of Posner (80) on attentional cueing, and the subsequent use of dot probe paradigms to evaluate the allocation of attention to competing stimuli with different motivational relevance (e.g. emotional faces: 11) and to drug related stimuli in addiction (65). Beginning in the early 2000s, a series of studies were conducted that, alongside with measuring the allocation of attention to smoking related cues through probe-detection tasks, also assessed the overt allocation of attention through eye-tracking (10; 28, 29; 71, 72). Unless otherwise stated, in the rest of our review we concentrate on the overt attention bias as assessed through the tracking of gaze.

**Figure 1.**
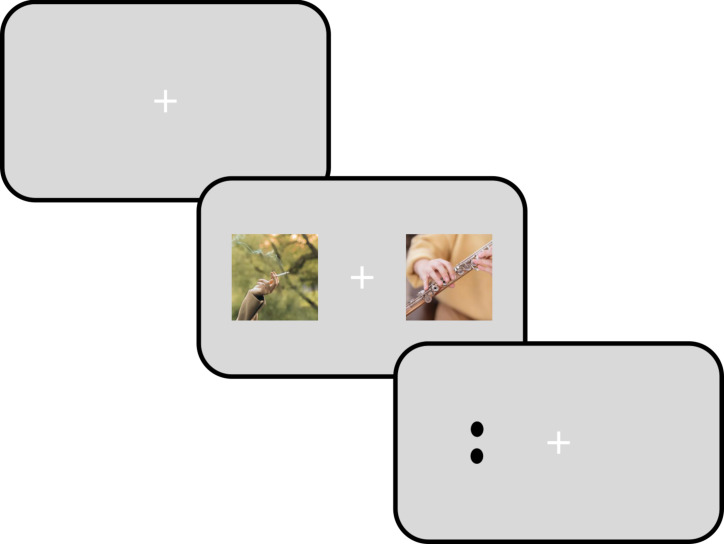
Schematic representation of the general paradigm used to test whether smoking-related content cues overt attention. Observers fixate centrally until two images, left and right of fixation, are presented, usually for one or two seconds. One of the images is related to smoking (unpredictably left or right, here left) whereas the other image is unrelated. The observer is free to move the eyes between the two images during their presentation. Commonly, a visual probe stimulus is also presented after the images, randomly left or right of fixation, in this case the two black dots. The observer is then asked for a speeded discrimination of the probe stimulus (e.g. indicating the number of dots) or to look towards it. Attentional cueing by smoking-related content is evidenced by oculomotor measures (e.g. longer dwell time on the smoking-related image) and by manual response times to the probe (i.e. relatively faster responses when the probe appears on the same side as the smoking-related picture).

A schematization of the general paradigm can be seen in Figure 1. Notice that one of its main features is the competition between two images, one smoking related and one smoking unrelated. Attentional allocation can be evaluated through oculomotor indexes, such as the percentage and latency of first saccades, dwell time or number of fixations on the smoking related picture, and through the manual response time advantage for probes presented on the side of the smoking related image. The most common finding that emerged in those studies was that smokers showed oculomotor signs of enhanced salience for the smoking related pictures, particularly in the form of longer dwell times on the smoking-related images. This effect is however mediated by both general characteristics of the observers, in terms of how long and how much they have been smoking, and by their state at the time of testing, e.g. their level of deprivation and/or craving. This parallels the results of studies that assessed the allocation of attention to stimuli by measuring only manual reaction times to a dot probe, which also indicated that the attentional bias towards smoking-related cues was possibly specific to some populations of smokers.

Rather counter-intuitively, the evidence seems to suggest that the bias towards longer fixation times on smoke-related pictures might be stronger in individuals with lower levels of dependence (72), similar to what emerged in the studies that investigated the attentional bias by means of manual reaction times to the probe, such as the one by Hogarth and colleagues (41). They found that the attentional bias strength followed an inverted-u shaped function of the number of cigarette smoked per day, with a peak attentional bias for smokers that smoked between 10 and 15 cigarettes/day and weaker biases for heavier and lighter smokers. Limited to the case where they used relatively short (500 ms) image presentations, Bradley and colleagues (12) also found the attentional bias, measured by means of manual response times, to be stronger for smokers that had attempted more often to quit smoking and that were smoking less often. There is no univocal interpretation for the stronger attentional cueing effect in light smokers, and in fact a more recent study (100) found an equal bias towards fixating smoking-related over neutral cues in dependent and non-dependent smokers. This was paired, in a slightly different paradigm, with an even stronger tendency to break the instructed fixation to look towards peripheral smoking-related cues in dependent smokers compared to nondependent smokers. The latter result was interpreted as indicating that a deficit in inhibitory control on the addiction-related stimuli emerges in later phases of substance use. One suggestion to explain a possibly stronger bias in less addicted individuals has been that the incentive associated with tobacco loses in relevance as the addiction becomes established (24). In this framework, when addiction progresses, smoking becomes a habit, smoking behavior becomes automatic and motivational aspects become less relevant. Another possibility is that as smokers become more and more addicted, their conditioned attention orienting response becomes more and more narrowly tuned, e.g. to the specific brand of tobacco product that they use, so that their orienting response to arbitrary smoking-related cues decreases (41). Finally, it could be that a history of repeated quit attempts, which Bradley and colleagues (12) found to be associated to lighter smoking, might increase the reward associated with smoking cues.

Alongside the observers’ history of smoking and quit attempts, the current state of the observer is bound to modulate the attentional bias, and one factor that likely promotes the attentional bias is the level of craving. Field and colleagues (28) indeed found that the bias was enhanced when smokers were nicotine deprived. Further studies showed that the bias increased after drinking a moderate dose of alcohol (29), but was reduced after 15 minutes of physical exercise (96). At least one report indicated that the tendency to preferentially fixate smoking-related cues can be reduced by transcranial alternating current stimulation over the dorsolateral prefrontal cortex, coupled with an attentional bias modification procedure, whereby observers are trained to detect probes that appear consistently on the side of the neutral cue (73). The administration of pramipexole, a dopaminergic agonist, can also lead to a reduction of the attentional bias, assessed as the proportion of first fixations on smoking-related cues when paired with non drug-related cues (33).

Notice that while smokers show an enhanced attention bias towards smoking cues compared to nonsmokers, they do not show an enhanced attentional bias for smoking-unrelated aversive stimuli, ruling out that a general change in saliency processing is responsible for the bias towards smoking cues in smokers (54). While the bias is specific for smoking-related cues, it also extends to images related to e-cigarettes (62), which might have implications for the regulation of e-cigarette advertisement. Finally, the individual strength of the attentional dwell time bias towards smoking related cues was found to be associated both with subjective craving and with the level of activation in a set of brain areas connected to addiction, as evidenced in a separate fMRI session (44).

Some recent studies have investigated the attentional bias to smoking cues using paradigms that differ partially from the original image-pair paradigm (Figure 1). For instance, Correa and Brandon (20) evaluated the fixation pattern of smokers when viewing images that contained both a smoking-related and another appetitive cue (e.g. a scene with a hand holding a cigarette next to a food item). While assessing the attentional bias, they exposed their observers to in vivo appetitive stimuli (e.g. observers held an actual package of cigarettes in their hand during testing). Although their results confirmed that smokers have a bias to fixate smoking-related cues, this was actually reduced when observers were exposed in vivo to the tobacco stimulus. Possible explanation for this result include a reduction of the appetitive value of the image once the in-vivo stimulus is available, or the fact that the in-vivo stimulus outcompetes the screen one for the observer’s attention. Haass-Koffler and colleagues (37) instead used a display with three stimuli: a smoking-related cue, an alcohol-related cue and a neutral cue. Their observers, people who both drank alcohol and smoked, showed an equal bias towards looking at the smoking- and alcohol-related cues compared to the neutral cue. The same bias emerged in the time the observers tended to spend interacting with in vivo alcohol and tobacco products, as opposed to water, when tested in a separate session within the context of a bar environment.

A difficulty in directing gaze away from a smoking related cues has been observed in an antisaccade paradigm (25). Here observers were shown smoking-related, alcohol related or neutral cues left or right of fixation, and had to execute a saccade towards the opposite location (antisaccade). Even light, nondependent smokers showed a larger tendency to make erroneous prosaccades to the smoking-related cues, compared to alcohol related cues and neutral cues. Observers who had never smoked instead were equally likely to make prosaccades towards neutral, alcohol related and smoking related cues, confirming that attentional biases emerge early in nicotine addiction (72).

When the attentional bias is measured by means of manual reaction times to the probe presented after the cues, different levels of cue duration can be used to investigate the time course of the bias. In particular, relatively short cue durations can be used to investigate whether attention is immediately captured by smoking-related cues in smokers. Unfortunately the results obtained with short cue presentations are not univocal. For instance Bradley and colleagues (9) found the attentional bias for cues presented for 200 ms, but a subsequent study only found a nonsignificant trend (8). Moreover, as we mentioned, Bradley and colleagues (12) found that the effect at 500 ms presentation was modulated by the number of quit attempts, whereas the effect was present equally for all smokers after 2000 ms presentation.

When investigating the attentional bias by means of eye movements, a measure of the initial capture of attention by smoking-related cues has been obtained by evaluating which of the two pictures observers look at first. Unfortunately, the results need to be taken with caution. While a majority of studies found a higher than chance proportion (generally between 53% and 55%) of initial saccades directed towards the smoke-related image, in the conditions that produced the enhanced dwell time (10; 28; 71, 72; 96), in one study the bias in the initial orienting of gaze was not significant. It should be noticed however that in this study the proportion of first saccades directed towards the smoking cue was 54.44% which is numerically comparable to what was observed in other studies (e.g. 29). Generally speaking it appears that the initial fixation bias is less reliable than the dwell time bias (22), which means that dissociations between the two indexes could potentially be due to measurement artifacts.

One final aspect that needs to be considered is that finding an attentional bias towards smoking-related stimuli in smokers does not necessarily imply that this bias is due to addiction. In fact, even never-smokers can show signs of attentional capture by smoking-related pictures when attention orienting is measured both through manual response times in visual search tasks (77), and by using the Late Positive Potential in ERPs (23). The results of the latter study also suggest that the enhanced saliency might be due to different mechanisms in smokers and never-smokers, given that smokers rate smoking-related images to be pleasant, whereas the never-smokers rate them as being unpleasant.

The paradigm used by the studies that we just described (Figure 1) has undoubtedly several desirable properties, including the fact that the stimuli can be controlled for low-level saliency, the oculomotor indexes can be identified without controversy, and the results can directly be compared to the manual response time indexes of attention allocation. Moreover, the situation of being exposed to two cues, symmetrically placed and similar in low level visual content, is most likely to reduce the measurement noise and highlight even the slightest bias in attentional allocation. It is however also a relatively artificial situation, and while its sensitivity might be desirable, one could question whether the enhanced salience that emerges between image pairs, would be detectable in more naturalistic situations as well. It also is not obvious what advantage the use of eye-tracking in such a paradigm offers over the assessment of attention by means of manual reaction times to a probe. On one side using eye-tracking provides a more dynamic representation of the allocation of attention, as the attentional bias can be tracked continuously within a trial as the exploration of the competing images progresses, on the other side measuring the attentional bias by means of manual responses makes the acquisition easier, for instance in the context of online studies. In the next section we describe another class of studies that specifically investigated the saliency of smoking related cues while smokers freely explore visual content. This research program can definitely be approached in a more straightforward way using eye tracking.

## Free exploration studies

There are different degrees to which the setting for an eye tracking study can be naturalistic, ranging from simple scene exploration on a two dimensional screen, to mobile eye tracking captured while observers go along their daily activities in their daily environment. The latter is probably not the best solution if one wants to study the way smokers and nonsmokers interact with smoking related cues. On one hand the availability of smoking-related items might differ in their daily environment, on the other hand such material might be altogether very rare for nonsmokers, depending on whether the local legislation allows for the advertisement of tobacco products and/or mandates that they are sold in dedicated shops, and depending on how widespread smoking in public is. Nonetheless, a few attempts have been made at verifying how tobacco addiction alters the individual patterns of ocular exploration in smokers within scenes, videos, virtual reality and controlled environments.

A first example is the study by Bonitz and Gordon (7). They measured eye movements while observers explored scenes presented on a computer screen. Within the scenes they inserted scene incongruent objects (e.g. a wrench on a food plate) and smoking related objects (e.g. a lighter). For comparison, in other versions of the same scene, the objects were swapped with scene congruent items (e.g. a fork) or smoking-unrelated items (e.g. a pack of chewing gums). The results of the study were consistent with the findings obtained with image pairs in the previous studies we discussed, i.e. the smokers spent more time fixating the smoking-related objects compared to the smoking-unrelated ones, which was not the case for nonsmokers. This bias was however less prominent than the bias that both smokers and nonsmokers showed towards fixating objects incongruent with the scene semantics, which is a known effect although its exact interpretation is beyond the scope of the present review (see 39; 97).

While static scenes are a more complex stimulus relative to isolated objects, they lack the dynamic aspect that characterizes our daily experience. The next step in this sense is the approach taken by Lochbuehler and colleagues (60), i.e. progressing from static scenes to video stimuli. They tracked the gaze of smokers and nonsmokers as they watched a segment of a Hollywood movie that contained scenes where the characters were smoking. The results confirmed the enhanced saliency for smoking material in smokers, demonstrated by the fact that smokers tended to look earlier, more often and for a longer time at smoking cues within the movie. In a similar vein, Lochbuehler and colleagues (58) demonstrated that children aged 10 to 13 who had at least one smoking parent made more and longer fixations to smoking-related material embedded in movies. Understanding how observers pay attention to smoking-related stimuli in movies might also be relevant for applied research, given that health warning messages could be inserted in movies or TV programs which contain smoking-related scenes, in order to mitigate their potential for promoting smoking. Indeed, a recent study (48) suggested that warnings should be presented prior to the smoking scene and not just concurrently, because the smoking cue reduces the time the observers spend looking at the warning, which in turn might modulate the effectiveness of the warning itself.

While videos incorporate the dynamic aspect of our everyday environment, and already pose additional challenges when eye movement data are evaluated, because pursuit occurs when observers fixate moving objects (1), watching a movie is still far from representing the way we commonly explore our environment, especially because the viewer sees the environment through the viewpoint of the camera decided by the movie maker. This problem can be mitigated by resorting to virtual reality. Gamito and colleagues (34) had observers navigate a three-dimensional simulated environment displayed on a computer screen, while they tracked their eye movements. Once again, the attentional bias was detected, as smokers proved to look more often to smoking-related cues compared to nonsmokers.

The observers in the study by Gamito and colleagues (34) could not use their body to navigate the environment, they still saw it through a fixed computer screen. To achieve a natural navigation of the environment, either eye tracking coupled with immersive virtual reality or mobile eye tracking are needed. This last step towards natural viewing was taken by Baschnagel (5), who had his observers wear a mobile eye tracker as they walked in an office environment that contained two smoking-related objects: an actual pack of cigarettes on a table and a poster depicting an actor smoking. The fact that smokers made a significantly (in fact more than three times) larger number of fixations to the smoking cues relative to nonsmokers confirmed that the attention bias that smokers have towards smoking cues is not a laboratory-only phenomenon, but is likely a tendency that extends to real-life situations. Another notable attempt at investigating eye movements by smokers and nonsmokers in a natural environment using mobile eye tracking is the pilot study by Bansal-Travers and colleagues (4), who tested observers as they walked into a convenience store to buy either a candy bar or a candy bar and a package of cigarettes. Albeit preliminary, their results seemed to indicate that a considerable proportion of observers looked at the wall where tobacco products were displayed, even if their task did not involve buying cigarettes.

All in all, the studies reviewed so far indicate that in general smokers show oculomotor signs of enhanced saliency for smoking-related cues, both in laboratory settings and in settings that are more akin to real-world situations. This however is not the only line of research that has used eye tracking to investigate smoking-related content. Often studies were conducted that had a more applied approach and used eye-tracking to investigate how smokers and nonsmokers interact with visual material meant to either promote or dissuade the use of tobacco. In the next section we delve into this line of research.

## Eye tracking applied to tobacco advertisements/warnings

The first study that investigated eyetracking with observers exposed to smoking-related material that we could trace dates from the late 1980s (31), and was precisely trying to answer the question whether consumers, in particular adolescents, actually read and later recalled the warnings that the United States Surgeon General at the time mandated to be inserted in advertisements for tobacco products, for instance in magazines. Interestingly enough, Fischer and colleagues (31) took a very naturalistic approach, using an early mobile eye tracking device that allowed the observers to freely browse through magazine pages that they held in their hands. Their results suggested that observers often skipped the warnings completely, and when they looked at them, it was for little time, often less then would have been required to actually read them. Not surprisingly, observers were also more likely to recall the content of the advertisement than the one of the warnings. The authors also already suggested the possibility that graphical, instead of text warnings could be more effective in competing with the graphics-based advertisements they were embedded in.

In the following years, researchers continued to investigate gaze behavior relative both to warnings in the context of advertisements and to warnings applied to packages of tobacco products (see 69 for a systematic review of the studies published until 2016). Some studies extended the original research by Fischer and colleagues (31) on viewing behavior relative to warnings embedded in advertisements. Krugman and colleagues (53) demonstrated that new warnings featuring a more direct message and an improved style of text, lead to more adolescent observers fixating the message and with a shorter latency. Moreover, they demonstrated that across observers a higher probability of recalling the text was related to both the number of fixations and to the mean dwell time on the warning. The fact that the text warnings commonly embedded in advertisements were not salient enough was again confirmed by Fox and colleagues (32), who measured the fixation patterns of adolescents on warnings embedded in advertisements for tobacco and alcohol products that were at the time printed in magazines in the United States. Strasser and colleagues (92) confirmed the hypothesis by Fisher and colleagues (31) that graphics-based warnings applied to advertisements would be fixated longer compared to text-only warnings, and showed that warnings that were fixated more often were also recalled better. The finding that graphical warnings are fixated more, compared to text-only warnings, has been confirmed in a more recent study that attempted at re-creating, at least partially, the context where point-of-sale advertisements are viewed (27). Crespo and colleagues (21) showed that verbal warnings inserted in tobacco advertisements were not more salient for smokers compared to nonsmokers, and that new warnings were not more salient compared to the ones the observers were commonly exposed to. Text warnings attract little overt attention when they are embedded in advertisements for smokeless tobacco products (50), and for products that are marketed as alternatives to cigarettes, such as nicotine-free cigarettes (59), snus (45), and heated tobacco products (56). Text warnings are also weak at competing for overt attention against price promotion labels on cigarillo packages (76) and against images of people in advertisements for e-cigarettes (91). Text warnings are also fixated less when embedded in advertisements for sweet or fruit flavoured e-cigarettes, compared to warnings in tobacco-flavored e-cigarettes (35). The fact that images advertising flavored e-cigarettes are more salient has been confirmed in adolescents who viewed the advertisements embedded in the image of a storefront (64). In this case, the participants’ bias to fixate the sweet/fruity/savory flavoured e-cigarette advertisements compared to the tobacco flavoured ones was also predictive of their reported willingness to try the different types of e-cigarettes. All in all, the fact that potential customers most likely pay little overt attention to text warnings associated with cigarette substitutes might contribute to them being perceived as relatively harmless.

Part of the motivation of the research on warnings inserted into advertisements, was to verify whether salient warnings would improve the reception of the warning message by the viewer, taking however into account the fact that, where legally allowed, advertisements needed to promote the product itself. In this sense, Peterson and colleagues (79) found evidence that although adolescents fixated warnings associated with health-related graphical images almost three times as often as text only warnings, and had better recall of the content of the warnings, the overall time that they spent looking at the advertisements was relatively unchanged. This suggested that images improved the reception of the warning message without repulsing the viewer from the ad itself.

**Figure 2.**
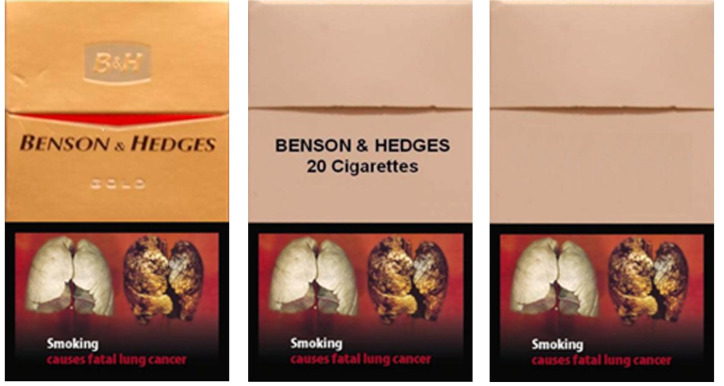
Example of GWLs applied to branded, plain and blank cigarette packages. Adapted from Maynard and colleagues (66).

Other studies instead focused on warnings applied to cigarette packages. Researching the saliency of warnings applied to packages through eye tracking has been crucial especially because in recent years different states have implemented policies specifically designed to enhance the saliency of those warnings. This included using both larger and graphical warning labels (GWLs), and to associate them with standardized plain packages which do not have brand information (Figure 2). Longitudinal surveys executed before and after the introduction of plain packages in Australia in 2012 (98) and in the United Kingdom and Norway in 2017-2018 (74) indicated that observers subjectively had the impression that warnings had become more effective after the introduction of plain packaging, and crucially that they noticed the warnings more.

This subjective impression has been shown to correspond to the measurable gaze patterns of potential customers. A direct comparison of gaze behavior to warnings based on graphics or on text indicated that GWLs are more likely to be fixated, assuming they take up at least 20% of the package surface (52). GWLs successfully compete for attention with the brand information (15), and unbranded packages enhance the saliency of GWLs in both smokers and nonsmokers (38; 88). There is however some evidence that even when all brand information is removed from the package, dependent smokers might still preferentially gaze at the plain part of the package, rather than looking at the GWL (66, 67; 75). Given that GWLs are designed to maximize bottom-up saliency, for instance by being embedded in high-contrast frames, the fact that smokers do not look at them seems to indicate that smokers actively (i.e. in a top-down fashion) avoid the warning picture. This active avoidance might emerge already in secondary-school daily smokers (67). Notice that stronger avoidance of GWLs on plain packages by daily smokers, compared to nonsmokers, was not found in a recent report (78). This might have to do with the fact that in this study the GWLs were placed at the top of the packages. Indeed, the position of the GWLs on the package seems to be relevant, since GWLs placed at the bottom of the package are fixated for shorter times (42), which might imply that they are easier to avoid. Similarly, Retzler, Shiraj and Retzler (85) found that the warning style mandated in the United Kingdom in 2016, which included GWLs and text warnings occupying the top part of the pack and branding in plain font, lead smokers to fixate more often the warning area relative to the rest of the package. A conflicting result in this regard was reported by Maynard and colleagues (67), who found that in daily smokers the bias to fixate the GWL as opposed to the branding area of the package was reduced with plain packaging. Generally speaking, the suggestion that smokers tend to avoid graphical warning is far from established. In fact, a recent study (89) found evidence of the contrary when comparing the time daily smokers spent fixating the image and the text in GWLs with different content. GWLs with images of death and disease, which in principle should have induced the maximum avoidance, instead induced longer viewing times on the image and less on the text warning, as compared to GWLs that had less arousing content, and that in principle should have produced less avoidance.

Beyond minimizing the possibility for the observer to evade threatening messages, one alternative solution in order to limit the possible impact of threat avoidance could be to convey the message using humor. For instance, an image of a cemetery with a missing grave indicated as “non-smoker area” would convey the message that “smoking kills” in a humorous way (6). A recent eye-tracking study suggested that humorous messages related to the health effects of tobacco and alcohol were fixated longer and more often, compared to threat messages (13). Particularly interesting in the latter study is the finding relative to refixations, which occurred less often for threat messages in smokers, compared to nonsmokers. This might be a sign of avoidance, but, crucially, humorous messages were refixated as often by smokers and nonsmokers, suggesting that avoidance was indeed circumvented by using humor.

Notice that GWLs are usually combined with text warnings on packages. Eye tracking has been used to investigate how the two types of warnings interact. Graphics might distract overt attention from the text. The extent to which this happens might depend however both on the content of the graphics and on the viewer. Regarding the content, it appears that disgust images reduce the time spent reading the associated text in adolescents compared to non-disgusting images (46), and the GWLs that are ranked as most effective possibly attract more sustained overt attention (70). Regarding the viewer, results are not univocal. One study (93) found that graphics distract overt attention from the text independently of whether the observer is a smoker or not. Another study (36) instead found that smokers have an even stronger tendency to look at GWLs over text compared to nonsmokers.

In spite of the apparent competition for overt attention, there is evidence that the graphical and text elements are processed together, because observers tend to spend more time looking at the text if it is unrelated to the GWL image (70), but a congruent GWL enhances the recall for the text warning (57). Notice however that this congruency advantage might fade with repeated exposure (61).

The results of eye tracking studies are also relevant to the broader question of whether campaigns to reduce unhealthy behavior should focus on threatening messages or rather provide coping information, for instance information on how to quit smoking, depending on the target of the campaign. Kessels and Ruiter (47) found that while nonsmokers spent more time looking at verbal threat messages, smokers spent more time reading coping messages.

Most of the research that applied the eye-tracking technique to topics related to tobacco regulations focused on health warnings embedded in advertisements for tobacco products or applied to product packages. A small number of studies focused instead on public service campaign advertisements that were meant to reduce smoking. Two studies compared fixation patterns on effective and ineffective public service advertisements (16; 87) containing both text and graphics. The effectiveness of the advertisements was established based on their public reception of the campaigns. The results seem to indicate that effective advertisements drew attention to the region of the advertisement image that was more relevant to the message. For instance, observers performed many fixations on the neck area of a former smoker that had received a tracheostomy. Conversely, ineffective advertisements induced more fixations on the warning text, which was taken as an indication of the fact that the message was less direct and required more time to be processed. Another study dealt with eye movements as observers interacted with a web-based campaign on the chemical constituents of cigarette smoke (51). The results indicated that a website with more pictorial graphics and that allows for interaction, for instance featuring elements that can be clicked, drew more overt attention compared to a web page containing only text. However, counterintuitively, such a website might not be as effective in conveying the message, given that it produced less recognition of the chemicals in a post-test. Jarman and colleagues (43) found that coping information, e.g. the number of a service to help people to quit smoking, could be added to other text elements, e.g. information about chemicals in the product and to graphics without reducing the time spent looking at the latter. They suggested that combining graphics with information and coping text is the best solution for framing an effective campaign advertisement. Wang and colleagues (99) instead investigated how the text associated with a public service advertisement depicting a scene involving a smoker and a nonsmoker changed the viewing pattern of smokers. If the text referenced harm to the observers, i.e. “smoking damages *your* body”, they were more likely to fixate on the smoker, compared to the case where the text referenced damage to others, i.e. “smoking damages *other’s* body”. While warnings applied to tobacco products can probably be aimed at maximizing the potential consumer’s aversion for the product itself, public health campaigns are also meant to convey information to the public. Albeit limited, the literature on eye tracking applied to public health campaigns stresses the importance of taking into account the interplay of text information and graphics, so as to produce the best combination of message saliency and volume of conveyed information.

Finally, we would like to underline the fact that the distinction between the different approaches that we have individuated when structuring our review is supposed to guide the reader but is not a strict one. One example that bridges the gap between the studies on the attention bias and the studies that are concerned with the effectiveness of tobacco advertisements and warnings is the one by Domaradzka and Bielecki (26). They showed that when smokers were exposed to an image of a pack of their preferred cigarette brand, paired with a package of a nonpreferred brand, they had more difficulties to disengage gaze from their preferred brand, suggesting that the graphical elements on a branded package produce an attentional bias in smokers.

## Conclusions and perspectives

Taken together, over the years the literature on eye tracking applied to smoking-related material has produced at least four main results that we can consider quite safely established:

1) Smokers show a tendency to fixate longer, and possibly look first at pictures depicting material related to tobacco and smoking, when they are paired with unrelated pictures, although, for reasons that are yet to be clarified, this effect might be limited to some classes of smokers, specifically light smokers, and might be modulated by the level of craving that the observer experiences at a given point in time.
2) This attentional bias extends from this rather artificial situation where two unrelated images are paired side-by-side, to conditions where the observer freely explores scenes, videos or the environment.
3) Graphics warning labels applied to tobacco product packages (or advertisements) are more likely to attract overt attention compared to text-only warning labels.
4) Plain (unbranded) packages enhance the saliency of warning labels.

We would however like to point out some unresolved questions and point to ways in which the field of research could potentially progress.

A first issue that we discussed is related to the possible tendency to avoid looking at GWLs in daily and heavy smokers, compared to light smokers or nonsmokers. If we consider studies that directly compared observers of different smoking status, we see evidence for avoidance in daily or heavy smokers in some studies (66; 75), one study reported no correlation with number of cigarette smoked per week (85), other studies failed to show a differential gaze behavior between smokers and nonsmokers (42; 78), one study found stronger avoidance in light smokers compared to heavy smokers (63). In fact, the idea that smokers avoid GWLs has been questioned altogether (89). As we mentioned, part of the discrepancy between the studies might have to do with the relative saliency of the GWL and to its placement and relative size on the package. Larger warning labels, placed at the top of the package, might be more difficult to evade. Further research would be needed to investigate how bottom-up and top-down contributions to overt attention interact to determine gaze behavior in different groups of observers.

A second issue, connected to the previous one, is related to the degree to which the characteristics of the observers or their attitudes determine the way they look at warnings. Groups identified in terms of age and degree of smoking addiction have been extensively tested, but especially when it comes to evaluating the effects of warnings that convey medical facts, other aspects, such as culture and health literacy (82) might play a role. Furthermore, research on the effectiveness of warnings suggests that observers are more likely to notice and use the information included in a warning if they are actively looking for it (3). At the same time the effectiveness of the warning is stronger if it confirms the beliefs of the observer (101). Indeed, the goal of the observers when viewing an advertisement might determine the way they explore it with their eye movements (40). It seems thus important to evaluate how the beliefs about the health risks associated with smoking held by the observers, and their modification brought about by public information campaigns or their social environment, modify the overt attention they dedicate to warnings applied to tobacco products. The prediction is that a positive feedback should ensue, whereby observers become more and more likely to notice anti-smoking warnings as they become more aware of the dangers associated with smoking.

A third issue, which applies both to studies that investigated the attention bias towards smoking-related cues and gaze towards tobacco advertisements and warnings, is the question as to whether gaze behavior is predictive of the attitude towards smoking and of the smoking behavior itself. The results of studies that measured both the attentional bias and smoking-related behavior are mixed. One study (37) found a that the bias towards overtly attending to smoking-related material was mirrored by a bias to spend more time interacting with tobacco products in a bar environment, but did not investigate whether the individual strength of the two phenomena were predictive of each other across observers. In fact, another study (22) failed to show an association between the attentional bias and the propensity of individual observers to smoke immediately after testing. The clinical relevance of the attentional bias in addiction in general, i.e. considering other addictive substances and methods of measuring the attentional bias, is far from established in the first place. A meta-analysis of 68 studies in 2009 already pointed to the fact that the relationship between the attentional bias and craving was relatively weak in general and particularly for tobacco and alcohol compared to other addictive substances (30). More recently, in a dedicated review article Christiansen and colleagues (17) pointed to the inconsistency both of the evidence that the attentional bias predicts relapse and of the evidence that attempts at modifying the attentional bias can have long-term effects on smoking behavior.

In the case of advertisements and warnings, it has been proposed that attention to the message is the first in a chain of information processing steps that lead to comprehension, recall and finally purchase or consumption behavior (68; also see 101). A meta-analysis on the effectiveness of warning labels (81), confirmed that salient warning messages enhance attention, but, specifically in the case of warnings that are meant to promote moderation or cessation of a product use, the meta-analysis also revealed that the effectiveness tends to decrease along the chain. Thus, a warning which is effective at capturing attention might not necessarily be as efficient to induce the viewer to smoke less or to quit smoking. While a few studies investigated whether gaze behavior was predictive of recall for warning messages, less research was dedicated to its relation to personal attitudes towards smoking, and the results are mixed. On one side there is some evidence that youth observers that gazed longer at GWLs also reported to be less likely to start smoking (15). Another report however failed to show a connection between the time spent looking at the warning label providing information on toxic chemicals in tobacco smoke and the intention to quit smoking (83). More research on the issue is necessary also in the light of recent evidence suggesting that both subjectively reported avoidance and reactance, i.e. the subjective experience of threat, can be dissociated from overt attention to health warnings within specific contexts (90).

But even attitudes towards smoking are only a step in the chain that leads to smoking behavior. The ideal final goal should be to measure the possible relation between overt attention to warnings and smoking behavior long term and beyond the lab setting. This would be best done through longitudinal studies that survey the evolution of the smoking behavior over longer periods of time. While this would be a time and resource-consuming solution, given that this question seems to be crucial to the social implications of this field of research, it appears necessary to expand the evidence in this respect.

Our final suggestion for the development of this field of research somewhat ironically brings us back to the oldest work that we reviewed, i.e. the pioneering study by Fischer and colleagues on gaze behavior while viewing advertisements that included warnings in magazines (31). The authors took an effort to test oculomotor behavior in a situation that was as ecologically valid as possible, having observers look at pages of an actual magazine while wearing a mobile eye tracker. With the exceptions of the study by Baschnagel (5), who investigated the attentional bias towards smoking material as observers moved freely through a room, and of the study by Bansal-Travers and colleagues (42016) who measured eye movements as observers shopped for tobacco products in a convenience store, almost invariably research on eye movements applied to smoking-related material has been conducted using static eye tracking and stimuli presented on a screen.

One possibility would be the one of conducting experiments where tobacco-related stimuli are viewed, and eye movements are measured, within the context of an immersive virtual reality setup, which is becoming an increasingly common testing environment (18). In a real complex environment, GWLs are probably not only competing for the observer´s attention against the brand information within the package, but also against a whole environment cluttered with objects, and it is an open question how this modulates the interplay of bottom-up and top-down contributions to gaze orienting. But in recent years mobile eye tracking has become both more accurate, affordable and widespread, well suited to testing even older adults while they execute their daily activities (2; 104), and the analysis of mobile eye movement data is becoming less and less cumbersome, given that the processing of scene videos can be automatized using computer vision techniques (e.g. 95; 102). It seems desirable that the next step in the investigation of how smokers and nonsmokers gaze at GWLs on cigarette packages should involve measuring oculomotor behavior as observers view and possibly manipulate actual cigarette packages, ideally embedded in an environment that reproduces the one where they are most likely to be observed, like the shop counter used by Bansal-Travers and colleagues (4). Measuring gaze in natural viewing conditions will be even more relevant when investigating overt attention to GWLs applied to the devices used to consume tobacco products and not only to the packages. For instance, it has been suggested that the position where a GWL is placed on a water pipe does not determine the amount of overt attention it attracts in a screen presentation (49). However, the warning position might make a huge difference when the observer manipulates the water pipe in real life. Given the omnipresent eye-hand coordination in daily activities (e.g. 55) one could expect that warnings placed near the hose might be gazed at more often when actually smoking a water pipe. In principle, even the mere fact of testing observers within the context of a study for which they volunteered could provide a biased representation of their spontaneous oculomotor behavior when viewing warnings or advertisements. Within the context of a study, observers might set themselves the goal of exploring the material, a goal that they might not have in real life and that might influence their exploration pattern (40). In the future, pervasive gaze sensing technology could provide the option to monitor gaze by people in a real-world environment and without their knowledge (14). This of course poses nontrivial issues of privacy and ethics, but would be the final step towards ecological assessment of observers’ attention towards smoking-related material

In conclusion, both tobacco smoking as a health and policy concern, and eye tracking applications to smoking are bound to remain relevant in the foreseeable future. We argue that adopting the most advanced approaches for monitoring gaze to investigate smokers’ gaze orienting in a naturalistic environment will significantly improve the ecological validity of the research on the effectiveness of health warnings, particularly as new tobacco products, messages, package outlines and images are introduced.

### Ethics and Conflict of Interest

The author(s) declare(s) that the contents of the article are in agreement with the ethics described in http://biblio.unibe.ch/portale/elibrary/BOP/jemr/ethics.html and that there is no conflict of interest regarding the publication of this paper.
